# Commentary on “Pembrolizumab in gestational trophoblastic neoplasia: systematic review and meta-analysis with sub-group analysis of potential prognostic factors”

**DOI:** 10.1016/j.clinsp.2025.100651

**Published:** 2025-04-24

**Authors:** Shubham Kumar, Rachana Mehta, Ranjana Sah

**Affiliations:** a Center for Global Health Research, Saveetha Medical College and Hospital, Saveetha Institute of Medical and Technical Sciences, Saveetha University, Chennai, India; b Clinical Microbiology, RDC, Manav Rachna International Institute of Research and Studies, Faridabad, India; c Department of Paediatrics, Dr. D. Y. Patil Medical College Hospital and Research Centre, Dr. D. Y. Patil Vidyapeeth (Deemed-to-be-University), Maharashtra, India; d Department of Public Health Dentistry, Dr. D. Y. Patil Medical College Hospital and Research Centre, Dr. D. Y. Patil Vidyapeeth (Deemed-to-be-University), Maharashtra, India; e Medical Laboratories Techniques Department, AL-Mustaqbal University, Babil, Iraq

Dear editor,

We would like to commend the authors of the manuscript titled “Pembrolizumab in Gestational Trophoblastic Neoplasia: Systematic Review and Meta-Analysis with Sub-Group Analysis of Potential Prognostic Factors” for their comprehensive systematic review.^1^ The study provides evidence on the use of pembrolizumab in the management of high-risk Gestational Trophoblastic Neoplasia (GTN), especially in chemoresistant or relapsed disease cases. The meta-analysis provides convincing arguments supporting the treatment's effectiveness and contains unique information useful for practitioners managing this complex disease. Nonetheless, we would like to make some comments on possible methodological refinements, as well as further analyses that could make the conclusions stronger.

The authors have assessed the quality of studies using the Newcastle-Ottawa Scale (NOS) which is commendable. The incorporation of GRADE certainty of evidence is commendable. Nonetheless, conducting a sensitivity analysis based on the study quality of included studies may reveal some information regarding the impact of study quality on the results. This type of analysis would enable the authors to determine whether lower-quality studies are disproportionately inflating the pooled estimates or biasing the results. The authors could eliminate high-risk of bias studies and in turn, systematically try to test the evidence for the robustness of the results with different quality levels.^2^ This is particularly important in the context of the findings which is their estimate of the reliability of the results. This is especially significant in the case of case reports and case series which have more methodological flaws relative to RCTs.

Despite the authors appropriately using a funnel plot to analyze publication bias, we recommend other techniques such as DOI plots of the LFK index for a more thorough evaluation of publication bias. This is more relevant in the case of proportional meta-analysis. The funnel plot can be ineffective with small study effects or publication bias when the level of asymmetry is modest. While the Trim and Fill method helps estimate and correct for lacking studies in meta-analyses, the LFK index effectively measures asymmetry.^3^ If these techniques were added to the analysis, it would help in gaining insight into publication bias and the accurate effect size, which would prove useful given the context of proportional meta-analysis.

Adding a Prediction Interval (PI) to the existing Confidence Intervals (CIs) would also improve the utility of the manuscript.^4^ Particularly in studying uncommon conditions such as GTN, A P1 would predict the effect of pembrolizumab on future studies. While the confidence interval gives the plausible values of the population effect, the prediction interval uses both within-study and between-study heterogeneity to provide estimates useful for clinicians who wish to anticipate future individual case outcomes. Because of the quote nature of the studies in the analysis, a PI would provide a larger treatment effect and more clinical relevance to the findings.

The authors performed a comprehensive subgroup analysis looking into age, histopathology, lines of chemotherapy, and interval to start immunotherapy. This is a great addition, as it assists in discernible heterogeneity and offers better specificity regarding the treatment effects. Nevertheless, future research might include some other prognostic features like molecular markers or immune signatures that would help clarify the scope of pembrolizumab's effectiveness. It would be fascinating to investigate the extent to which responders and non-responders differ at the molecular level, helping design personalized immunotherapy strategies for GTN.

## Ethical approval

Not applicable.

## Authors’ contributions

SK, RM, RS, critically and provided comments on methodological aspects, SK and RS have written the edited the draft.

## Funding

No funding was received for this research.

## Declaration of competing interest

The authors declare no conflicts of interest.
